# Supplementary material for: Molecular and Functional Characterization of Different BrainSphere Models for Use in Neurotoxicity Testing on Microelectrode Arrays
Source: Cells. 2023 Apr 27;12(9):1270. doi: 10.3390/cells12091270 (PMC10177384; doi:10.3390/cells12091270)
Supplement: Supplementary file 1 [file cells-12-01270-s001.zip › Hartmann et al 2023_Supplementary Tables S1 to S3.pdf]

1. Supplementary Tables

Supplementary Table S1. Coatings used for neural differentiation of BrainSpheres generated with different neural induction protocols.

Neural induction protocol	Differentiation medium	coating	Order number and supplier
2D-NIM	CINDA+	0.1 mg/mL PLO[1] / 50 µg/mL laminin	PLO: #P4957, Sigma-Aldrich, St. Louis, MO, USA<br>laminin: #LN521, Biolamina, Sundbyberg, Sweden
	Electro	0.1 % PEI[2] / 50 µg/mL laminin	PEI: #181978, Sigma-Aldrich, St. Louis, MO, USA<br>laminin: #LN521, Biolamina, Sundbyberg, Sweden
GNEIB	CINDA+	25 µg/mL laminin	laminin: #LN521, Biolamina, Sundbyberg, Sweden
	Electro	0.015 mg/mL PLO[1] / 50 µg/mL laminin	PLO: #P4957, Sigma-Aldrich, St. Louis, MO, USA<br>laminin: #LN521, Biolamina, Sundbyberg, Sweden
Stemdiff	CINDA+	50 µg/mL laminin	laminin: #LN521, Biolamina, Sundbyberg, Sweden
	Electro	0.1 mg/mL PLO[1] / 50 µg/mL laminin	PLO: #P4957, Sigma-Aldrich, St. Louis, MO, USA<br>laminin: #LN521, Biolamina, Sundbyberg, Sweden

[1]PLO: poly-L-ornithine; [2]PEI: polyethyleneimine

Supplementary Table S2. Information about used compounds and their solvents.

Compound	CASRN	Order number and supplier	solvent	effect
Bicuculline	485-49-4	#0130, Tocris, Bristol, UK	DMSO	$GABA_A$ antagonist
Buspirone hydrochloride	33386-08-2	#HY-B1115, MedChemExpress, South Brunswick, NJ, USA	DMSO	5-HT1A agonist
Carbaryl	63-25-2	#32055, Sigma-Aldrich, St. Louis, MO, USA	DMSO	inhibits acetylcholinesterase
DL-2-amino-5-phosphonovaleric acid (AP5)	76326-31-3	#SC-201503, Santa Cruz Biotechnology Inc., Dallas, TX, USA	H_2O	NMDA antagonist

Emamectin benzoate	155569-91-8	#31733, Merck, Darmstadt, Germany	DMSO	$GABA_A$ and $GABA_C$ agonist
Haloperidol	52-86-8	#HY-14538, MedChemExpress, South Brunswick, NJ, USA	DMSO	D2 antagonist
L-glutamate	6106-04-3	#49621, Sigma-Aldrich, St. Louis, MO, USA	H_2O	Neurotransmitter
NBQX disodium salt (NBQX)	479347-86-9	#SC-222048, Santa Cruz Biotechnology Inc., Dallas, TX, USA	DMSO	AMPA antagonist
Picrotoxin	124-87-8	#SC-202765, Santa Cruz Biotechnology Inc., Dallas, TX, USA	DMSO	$GABA_A$ and $GABA_C$ antagonist
Trimethyltin chloride (TMT)	1066-45-1	#146498, Merck, Darmstadt, Germany	H_2O	Increases glutamate release
γ-Aminobutyric acid (GABA)	56-12-2	#A5835, Sigma-Aldrich, St. Louis, MO, USA	H_2O	Neurotransmitter

Supplementary Table S3. Primer sequences.

**Gene**	**Forward primer sequence (5′ → 3′)**	**Reverse primer sequence (5′ → 3′)**
ACHE	GTCCAGACTAACGTACTGCT	ATGCGATACTGGGCCAAC
ACTB	CAGGAAGTCCCTTGCCATCC	ACCAAAAGCCTTCATACATCTCA
AQP4	TGGACAGAAGACATACTCATAAAGG	GGTGCCAGCATGAATCCC
CHAT	GCCCATAGTATTGCTTCATGC	ACCAGCTGAGGTTTGCAG
CHRNA4	CTCGCGGGCTCTAGATG	CCACAGGAGAAGACGAACC
DLG4	CCAGAAGAGTACAGCCGATTC	CTTGGTCTTGTCGTAATCAAACAG
DRD2	ACGGCGAGCATCCTGAACTT	GCCGGGTTGGCAATGATGCA
EN1	CTGGGTGTACTGCACACGTTAT	TACTCGCTCTCGTCTTTGTCCT
FOXG1	TCCATCCGCCACAATCTGTC	CCCAGCGAGTTCTGAGTCAA
GABRA1	ACAACACTTACGCTCCAACA	TTTCGGGCTTGACCTCTTTAG
GAD1	GACCCCAATACCACTAACCTG	GCTGTTGGTCCTTTGCAAG
GFAP	CACTGTGAGGCAGAAGCTC	CCTCCAGCGACTCAATCTTC
GLS	CAGTTCCAAGATCATTAACAGCA	CGATTTGTGGGGTGTGTCT
GRIA1	CTTAATCGAGTTCTGCTACAAATCC	GTATGGCTTCGTTGATGGATTG
GRIN1	CTCCTGGAAGATTCAGCTCAA	GTGGATGGCTAACTAGGATGG
HOXA2	CACCACGTCTACGGGCAAGAAC	AGCTGCTGATGCTGACTTCTGA
HTR1A	TGTCGCTTCTCACAAACTCTC	CTTCTCCTCTTTCTCTCTGCTC
LMX1A	CGGGTCATCTTGGACAGGTTT	CAGAAGCAGCTCAGGTGGTA
MAP2	TGCCTCAGAACAGACTGTCAC	AAGGCTCAGCTGTAGAGGGA
S100B	CACATTCGCCGTCTCCATC	CACAAGCTGAAGAAATCCGAAC
SLC12A2	ACAAAGTTGAGGAAGAGGATGGC	CCTGATCTGCCGGTATGTCTTGG
SLC12A5	CCGATGACAATTACCCATGGA	CTGTCTACATCAGCTCCGTTG
SLC17A6	CCTCTACCCTAAATATGCTAATTCCA	TTGCTCCATATCCCATGACAT
SLC17A7	CAGAAAGCCCAGTTCAGC	ACAATAGCAAAGCCGAAAACTC
SYN1	GTGCTGCTGGTCATCGAC	CCACAAGGTTGAGATCAGAGAA
TH	GGTCTCTAGATGGTGGATTTTGG	TGCTAAACCTGCTCTTCTCC
TPH1	TGTCTCACATATTGAGTGCAGT	CCACAATTGGAAGATGTCTCC
